# Range area and the fast–slow continuum of life history traits predict pathogen richness in wild mammals

**DOI:** 10.1038/s41598-023-47448-3

**Published:** 2023-11-18

**Authors:** Jacqueline Choo, Le T. P. Nghiem, Ana Benítez-López, Luis R. Carrasco

**Affiliations:** 1 Department of Biological Sciences, National University of Singapore, Singapore, Singapore; 2 University of British Columbia, Vancouver, Canada; 3 Department of Biogeography and Global Change, Museo Nacional de Ciencias Naturales (MNCN-CSIC), Madrid, Spain

**Keywords:** Computational models, Diseases, Ecological epidemiology

## Abstract

Surveillance of pathogen richness in wildlife is needed to identify host species with a high risk of zoonotic disease spillover. While several predictors of pathogen richness in wildlife hosts have been proposed, their relative importance has not been formally examined. This hampers our ability to identify potential disease reservoirs, particularly in remote areas with limited surveillance efforts. Here we analyzed 14 proposed predictors of pathogen richness using ensemble modeling and a dataset of 1040 host species to identify the most important predictors of pathogen richness in wild mammal species. After controlling for research effort, larger species geographic range area was identified to be associated with higher pathogen richness. We found evidence of duality in the relationship between the fast–slow continuum of life-history traits and pathogen richness, where pathogen richness increases near the extremities. Taxonomic orders Carnivora, Proboscidea, Artiodactyla, and Perissodactyla were predicted to host high pathogen richness. The top three species with the highest pathogen richness predicted by our ensemble model were *Canis lupus*, *Sus scrofa*, and *Alces alces.* Our results can help support evidence-informed pathogen surveillance and disease reservoir management to prevent the emergence of future zoonotic diseases.

## Introduction

The majority of emerging infectious diseases of pandemic potential are zoonotic diseases^1,2^. Examples include the H1N1 influenza pandemic (1918), severe acute respiratory syndrome (SARS) coronavirus (2002–2004), Ebola virus (2013–2016), and the recent severe acute respiratory syndrome coronavirus 2 (SARS-CoV-2) that started in 2019^3^, whose origin is still being debated^4^. Bat species have been identified as the most probable wildlife origin for SARS-CoV-2^5^ with pangolins acting as intermediate hosts^6^. In turn, bats, non-human primates, and duikers have been reported as spillover sources of the Ebola virus ^7^. These zoonotic diseases have severe health, economic, and social impacts^8^. For example, the SARS-CoV-2 outbreak has resulted in more than 5.4 million deaths^9^ and a loss of 2.4 trillion US dollars in global gross domestic product^10^. Due to the huge financial and public health cost of zoonotic diseases, it is imperative to monitor zoonotic disease outbreaks^8^. A potential pathway is the development of predictive models that can identify wildlife species with a high risk of hosting zoonotic disease.

Species that harbor more pathogens are more likely to lead to zoonotic disease spillover when there is close human–wildlife contact, making them potential zoonotic disease reservoirs^2,11,12^. Hence, the monitoring and prediction of pathogen richness in host species is crucial to prevent future zoonotic disease outbreaks^11,12^. Previously identified predictors of pathogen richness in wildlife species include evolutionary history, species geographic distribution, species traits, and anthropogenic factors^11–16^. There is evidence that phylogenetically similar species have higher spillover risk^15,16^ and that certain taxonomic groups (i.e. Rodentia and Carnivora) harbor higher zoonotic pathogen richness^17^. In turn, species geographic distribution affects pathogen exposure^11,16,18,19^. Because species with greater geographic range area and altitude breadth are more likely to occupy different habitats, the chances of coming into contact with a variety of host species and fomites are higher, thus increasing their pathogen exposure^11,20^. Indeed, geographic range overlap with a high number of species has been reported to be positively correlated with greater virus richness^18,19^.

Fast-lived species are hypothesized to have higher pathogen richness^13^ and tend to be more resilient to human disturbance, allowing them to thrive in anthropogenic environments^21^. This allows them to make use of new available resources (e.g., anthropogenic food sources and declining competing species population due to hunting) and live in close proximity to humans. This leads to higher population density of fast-lived species, promoting the spread of many pathogens. Another hypothesis is based on the trade-offs between reproduction and immunity^13^. Fast-lived species invest more energy into reproduction, resulting in nonspecific and weaker immune responses to diseases^13^. For instance, fast-lived birds with shorter incubation periods have lower natural antibody levels^22^ and lymphocytes^23^. However, there is limited evidence that supports this claim in mammals^13^. A study on three wild rodent species found that fast-lived species have a smaller antibody response than slow-lived species^24^. However, Cooper et al.^12^ found that lower longevity (fast-living) in ungulates, carnivores, and primates was not correlated with lower white blood cell counts. Hence, the pathway in which life history traits influence pathogen richness may not only be a result of the reproduction and immunity trade-offs, but may also include how different traits affect pathogen exposure and persistence.

An alternative hypothesis suggests that slow-lived species have higher pathogen richness^13^. Species with longer life spans are hypothesized to encounter more pathogens and provide more time for pathogen multiplication and colonization^25^. Further, larger-bodied species provide more niches for pathogen establishment (e.g. greater surface area for ectoparasites)^25^ and have greater energy requirements, food intake, and thus, greater risk of foodborne pathogens^11,25^.

Anthropogenic factors such as livestock density and land-cover change can also affect pathogen transmission and host species’ energy allocation to immunity^26–28^. High livestock density facilitates pathogen transmission as it increases the risk of pathogen spillback from livestock to wildlife^28^. In turn, land-cover changes such as urbanization and conversion of habitats for agriculture can also result in increased human-wildlife contact and changes in resource availability^27,29^. For instance, urbanization results in the loss of habitats and resources, leading to poorer health conditions of wild animals^30^. The reduction in resources can subsequently lead to decreased energy allocation to immunity and increased pathogen richness^27^. Conversely, some species are able to take advantage of anthropogenic food sources (e.g. rubbish bins, plants from backyards and streetscapes, and domestic animals), allowing them to adapt to urban environments^29,31^. Urban and agricultural areas may also release contaminants into the environment which can directly affect wildlife health and immunity^27,30^.

Anthropogenic and intrinsic (species traits) predictors of pathogen richness have been examined in recent literature. For example, Gibb et al.^21^ found that there was higher wildlife host species abundance in agricultural and urban land-use. The relationship between pathogen richness and life history traits was assessed by Cooper et al.^12^, Kamiya et al.^11^, and Plourde et al.^15^, whose studies show that pathogen transmission and persistence is greatly affected by both intrinsic (e.g. species life history traits) and extrinsic (e.g. land-cover changes) factors^11,21,32^. Yet, the examination of the interactions between both extrinsic and intrinsic factors and the relative importance of each predictor for explaining pathogen richness have not been properly studied, hence limiting our understanding of the determinants of pathogen richness. To address this, we analyzed the extrinsic and intrinsic predictors of pathogen richness in mammal species collectively. The results of this study may provide a first indication of which species to monitor and further study. We used a dataset of 1040 host species and 2675 unique pathogens, applying an ensemble modelling approach (1) to identify the extrinsic factors and species traits that are associated with pathogen richness, (2) to assess their relative importance for predictive purposes, and (3) to identify the species and taxa with the highest predicted pathogen richness.

We analyzed the proposed predictors of pathogen richness in mammals using an ensemble model composed of bagged random forest and boosted regression tree models. Ensemble modeling combines predictions of multiple models, which reduces model uncertainty and results in a more reliable and accurate prediction^33^. Machine learning and ensemble modelling are not often utilized in understanding factors associated with wildlife pathogen richness and its prediction (but see^34^). Thus, the modelling approach in this study demonstrates an alternative, more powerful method compared to regression-based models that allows analyzing large datasets with potential interactions between the predictors. Based on previous research results, we hypothesize that species with higher reproductive rate have greater pathogen richness. Furthermore, we expect that greater urban and agricultural land-cover change within the geographic range of a species will lead to increased pathogen richness because the species will be more exposed to pollutants and domestic animals. Knowledge gained from this study may help inform pathogen surveillance in wildlife to prevent future zoonotic emerging infectious diseases and help shed light on the pathways by which these extrinsic and intrinsic factors affect pathogen richness.

## Methods

### Mammal pathogen data

We used the mammal pathogen database collated by Gibb et al.^35^. This database comprises information from four main sources (Global Mammal Parasite Database, Enhanced infectious Diseases Database, Host-Parasite Phylogeny Project dataset, and the host–pathogen data curated by Shaw et al.^19^), thus resulting in the most comprehensive mammal pathogen dataset to date, with 1109 wildlife host species and 2851 unique pathogens. After filtering for terrestrial mammals, we were left with 1041 host species (2675 unique pathogens). *Cercopithecus* *pogonias* was found to have missing predictor data and excluded from this study, leaving 1040 species.

### Predictors of pathogen richness

Predictors were proposed based on previous research and theories (Supplementary Table S1). Main predictors of pathogen richness identified by past research are species fast–slow continuum of life history traits^11–13,15,18^, species geographic distribution (range area, altitude breadth, number of species with overlapping ranges)^11,18,19^, and anthropogenic factors (land-cover change^21,27^ and livestock density^36,37^) (Supplementary Table S1). Greater sampling and research effort on certain species can result in increased discovery of pathogens. Sampling and research effort are influenced by multiple factors such as ease of sampling (accessibility of a species habitat and species abundance)^13^ and scientific interest (e.g. surveillance of rodent species for zoonotic diseases)^15^. Hence, we added a research effort variable in all our models to account for bias due to differing sampling and research effort^19,38^. Research effort in a disease context for each mammal species in our dataset was determined by the number of hits returned when searching for each mammal species name (binomial) and keywords “infect*” or “zoono*” or “disease*” or “bacteria*” or “virus” or “parasit*” or “pathogen*” or “epidemic” or “epizootic” from year 1950 to 2022 in the PubMed database^19^. The data on species fast–slow continuum of life history traits were obtained from the COMBINE database^39^. The COMBINE database includes life history trait data compiled from different studies and databases, and missing data were imputed using Random Forest^39^. Only species fast–slow continuum of life history traits that had less than 70% of the data imputed were used as predictors in this study. Life history traits may have some degree of correlation with each other. We performed a principal component analysis (PCA) to summarize the different traits into a two-dimensional space. Life history traits with right skew distribution were log-transformed prior to the PCA analysis. We used the original life history traits and the PCA axes to fit separate models and reported the results of both models. We decided to present both models to allow interpretation of the correlation between individual life history traits and pathogen richness before comparing it to using PCA axes that reduce potential collinearity between life history traits.

Land-cover change from 1992 to 2020 was calculated for each mammal species across their range. Global land-cover raster maps were sourced from the ESA CCI Land Cover and the EC C3S Land cover project^40^. Polygons of species’ extant geographic range were obtained from the International Union for Conservation of Nature (IUCN)^41^. The percentage of agricultural and urban land-cover for year 1992 and 2020^42^ were extracted for each species range and the percentage change in land-cover was calculated (% of agricultural/urban land-cover in 2020 minus % of land-cover in 1992). A positive value will indicate an increase in agricultural/urban land-cover while a value of 0 indicates no change in agricultural/urban land-cover from 1992 to 2020.

Number of species whose ranges overlap with the range of the target species (number of species with overlapping ranges) was calculated based on the number of species whose range has a 20% or more overlap with the target species’ range. This threshold has been identified as the minimum geographic overlap needed to facilitate viral sharing between hosts^18^. The total number of pigs, cattle, and buffalo were extracted from the Food and Agriculture Organization of the United Nations (FAO) Gridded Livestock of the World data^43^ and summed up across each species’ range. The total number of livestock was then divided by the species range area to obtain the livestock density for each species.

### Data analysis

We used bagged random forest and boosted regression tree models to identify predictors of pathogen richness. Research effort and species taxonomic order variables were added to account for research bias and taxonomic relatedness. Hyperparameter tuning was performed for both random forest and boosted regression tree models to determine the best model parameters to use based on the lowest root-mean-square error (RMSE). This is to optimize the models and select for parameters that give the highest prediction accuracy. Hyperparameter tuning for the random forest model was conducted to decide a suitable random subset of all predictors to be considered by the model at each split. This is represented by the *mtry* argument in the randomForest^44^ R function. The number of trees used in the random forest model was set as 500. For the boosted regression tree model, hyperparameter tuning for shrinkage, interaction depth, bag fraction, and optimal number of trees was conducted. The final models with the optimized parameters were used to determine predictor importance based on the lowest percentage increase in mean-square error if that predictor was excluded from the model.

Studies modelling pathogen richness mainly omit species that do not have pathogen data. However, the missing pathogen data is not random as more research effort is placed on certain taxonomic orders^13,17^. Furthermore, fast-lived species are more likely to be sampled as they are more abundant and live in closer proximity to humans as they adapt better to urban areas^13,38^. Hence, excluding species without pathogen data can misrepresent relationships, leading to false positives and bias^13^. To account for this, pseudo-zeros were added into the data.

Out of the known 5707 terrestrial mammal species^41^, we had complete predictors data for 5537 species. From this dataset, we used MatchIt package^45^ to select 1040 species with missing pathogen data based on the distribution of fast–slow continuum of life history traits of the species dataset for which pathogen richness data exist. We assigned a pseudo-zero to these species to obtain a balanced dataset of 2080 species. Non-negative and non-zero predictors with right skewness were log-transformed while the rest of the predictors were standardized (the mean was subtracted from the value and then divided by the standard deviation). Zero-inflated negative binomial mixed-effects models were fitted using the pseudo-zero datasets to estimate the effect of the predictors on pathogen richness after adjusting for the probability of false zeros with species family taxon as the random effect. Thus, the zero-inflated models considered taxonomic relatedness, research effort, and missing data when modelling the predictors against pathogen richness. The models were then checked for multicollinearity (VIF < 5).

We assessed the predictive performance of our models by randomly splitting the data into two sets, a training and a testing set. One third of our data was set aside for testing and to determine the prediction accuracy of the model while the rest of our data was used for model training. As the model has not seen the testing dataset before, the model’s ability to predict the testing dataset would be a better reflection of its prediction accuracy and prevent a high accuracy score due to overfitting. The model’s performance was determined using RMSE, which measures the average squared difference between predicted values and the observed values. A lower RMSE indicates better performance. Furthermore, as our data was randomly split into two sets, we ascertained whether the random split of the data would affect the models’ performance by conducting tenfold cross-validation for each model.

### Ensemble model prediction

Our ensemble model consists of the bagged random forest and boosted regression tree models. The zero-inflated model was found to perform poorly and was thus not included in the ensemble model. The predictions by the two models were averaged and weighted by the inverse of their RMSE^46^. We constructed two ensemble models: models using the original life history traits and models using the PCA axes. The ensemble models were used to predict pathogen richness of the 1040 mammals and the predictions were plotted against the observed pathogen richness.

We also used our ensemble models to predict pathogen richness data for mammal species with predictor information. Pathogen richness was predicted for 5443 out of the 5537 mammal species. The remaining 94 species could not be predicted by the machine learning models as they were from orders that were absent from the training dataset. Research effort was set as the average value for all species to account for research bias. Additionally, as order Proboscidea was suspected to be an influential point, an uncertainty analysis was conducted by excluding order Proboscidea from the data analysis and predictions.

## Results

### Principal component analysis (PCA)

The first two dimensions of the PCA explained 71–80% of the species fast–slow continuum of life history traits. The first axis (Comp.1) showed the trade-offs of species life history traits on the fast–slow continuum: with higher maximum longevity, age at first reproduction, female maturity, weaning age, and gestation length, there was smaller litter size and number of litters per year. The positive values on the first axis therefore showed slow-lived species while fast-lived species were towards the negative values. Conversely, the negative values on the second axis (Comp.2) showed species with greater neonate mass, and thus falling on the slow end of the continuum (Supplementary Fig. S1).

### Random forests

**Figure 1:**
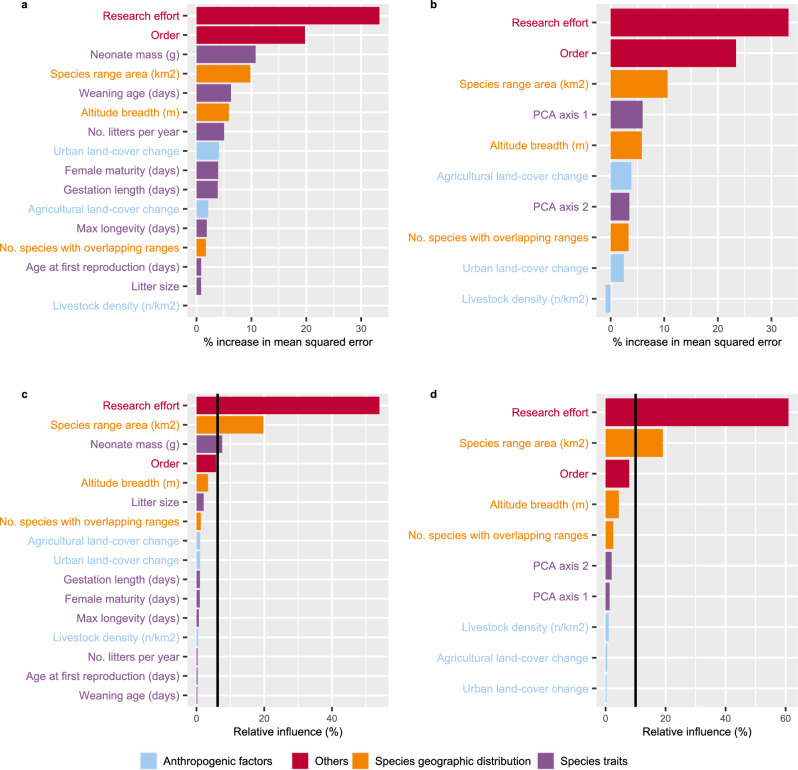
Summary of predictors importance in the random forest and boosted regression tree models. (**a**) random forest fitted with the original life history predictors; (**b**) random forest fitted with the PCA axes; (**c**) boosted regression tree fitted with the original life history predictors; (**d**) boosted regression tree fitted with the PCA axes. The percentage increase in mean squared error shows the increase in the model error (decrease in the model accuracy) if the variable is excluded. The relative influence shows the relative contribution of the explanatory variable to predicting mammalian pathogen richness. The vertical line shows the value of relative influence expected by chance (6.25% and 10%).

**Figure 2:**
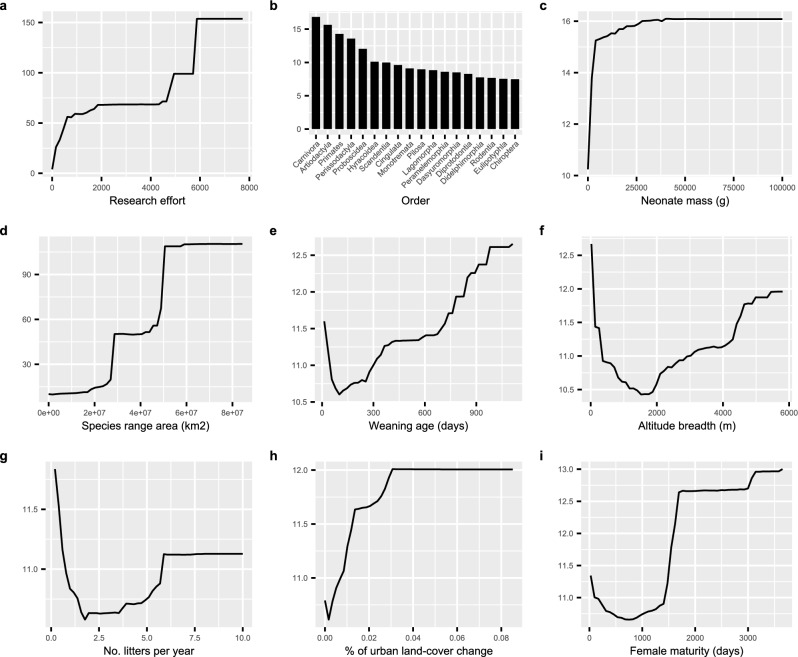
The partial dependence plots of the top nine predictors of the random forest model fitted with the original life history predictors.

**Figure 3:**
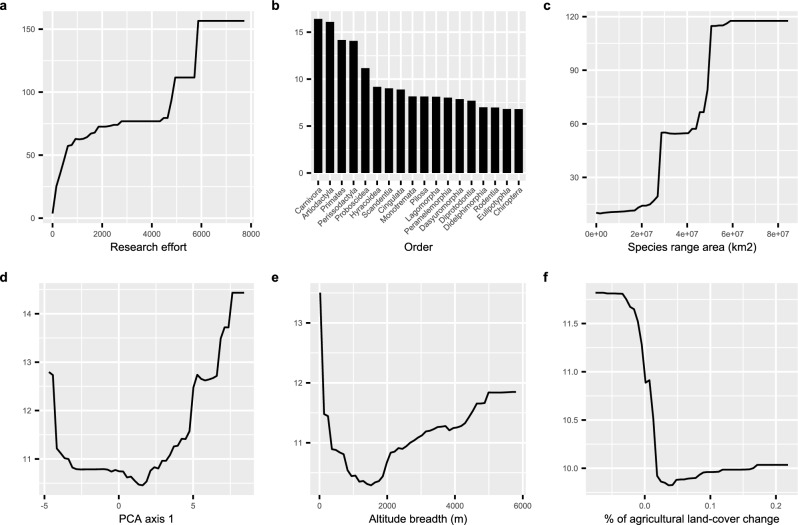
The partial dependence plots of the top six predictors of the random forest model fitted with PCA axes. The negative values on PCA axis 1 are more fast-lived species and the positive values are more slow-lived species.

The random forest model fitted with the original life history traits showed that research effort was the variable with the highest importance, followed by taxonomic order, and neonate mass (Fig. 1). When considering PCA axes as predictors of life history traits, this importance ranking changed to research effort, order, and species range area (Fig. 1). The partial dependence plots showed that research effort, neonate mass, and species range area were positively correlated with pathogen richness. Some fast–slow continuum of life history predictors (weaning age, number of litters per year, female maturity, and the PCA first axis) showed a U-shaped relationship where both very fast and very slow-lived species had high pathogen richness. Altitude breadth also showed a U-shaped correlation with pathogen richness. Pathogen richness increased with higher urban land-cover change but decreased with higher agricultural land-cover change (Figs. 2 and 3).

### Boosted regression trees

**Figure 4:**
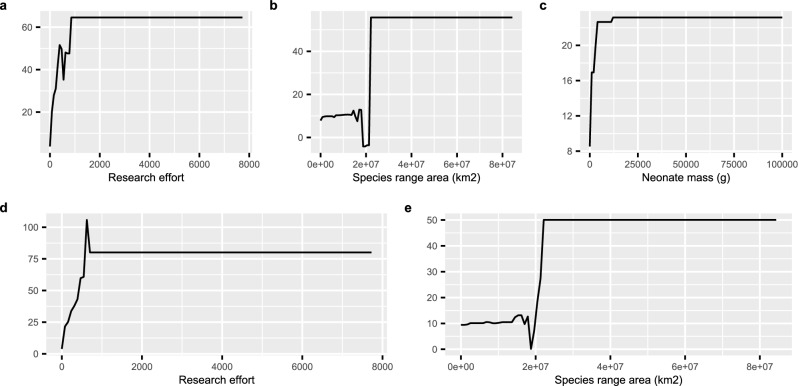
The partial dependence plots of the important predictors of the boosted regression tree models. (**a**–**c**) fitted with the original life history predictors; (**d**–**e**) fitted with the PCA axes.

The boosted regression tree model fitted with the original life history traits showed that research effort, species geographic range area, and neonate mass were the predictors with the highest importance (Fig. 1). Research effort and species range area remained important predictors also when using PCA-axes as predictors (Fig. 1). The partial dependence plots showed that both research effort, species range area, and neonate mass were positively correlated with pathogen richness (Fig. 4).

### Zero-inflated model

**Figure 5:**
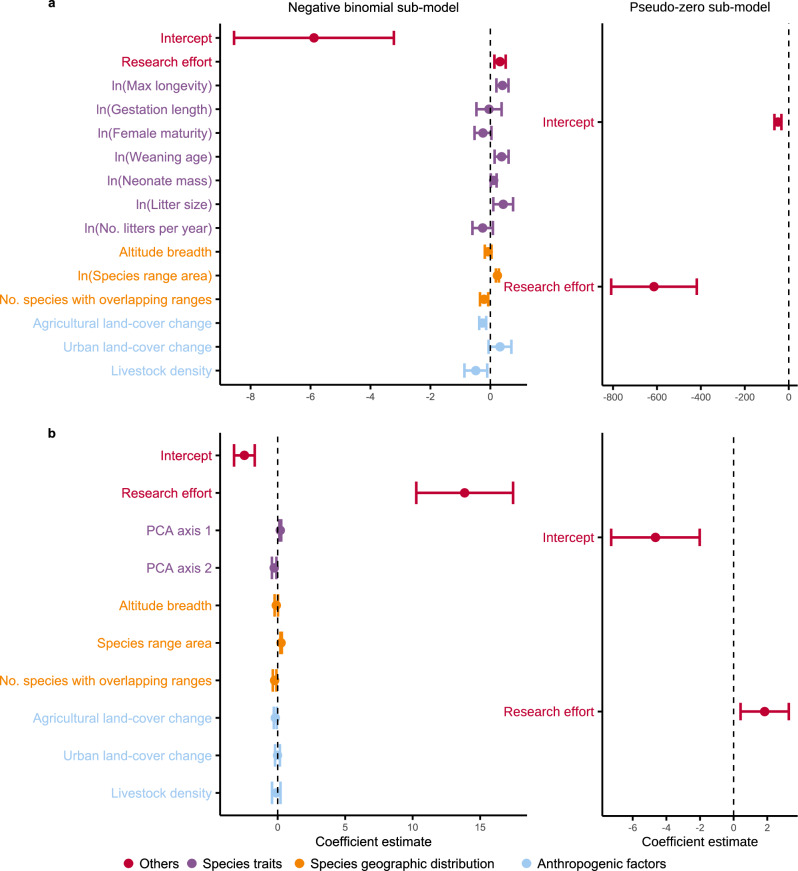
Results of the zero-inflated negative binomial mixed-effects models. Selected predictors with right skewness were log-transformed and the rest of the predictors were standardized. (**a**) fitted with the original life history predictors; (**b**) fitted with the PCA axes. The error bars show the coefficient estimates of the predictors at 95% confidence interval. The negative binomial sub-model shows the effects of the predictors on pathogen richness. The pseudo-zero sub-model (a binomial logistic model) shows the effect of research effort on the probability of false zeros.

The zero-inflated negative binomial mixed-effects model fitted with the original life history traits showed that species geographic range area, maximum longevity, litter size, and research effort were significantly positively correlated with pathogen richness (range area: *P* value < 0.01, estimate = 0.23; maximum longevity: *P* value < 0.01, estimate = 0.40; litter size: *P* value = 0.01, estimate = 0.43, research effort: *P* value < 0.01, estimate = 0.32). Agricultural land-cover change and livestock density were significantly negatively correlated with pathogen richness (agricultural land-cover change: *P* value < 0.01, estimate = − 0.26; livestock density: *P* value = 0.01, estimate = − 0.49) (Fig. 5 and Supplementary Table S9). Max longevity, litter size, and livestock density had the largest effect sizes.

When considering PCA, the PCA first axis, research effort, and species range area were significantly positively correlated with pathogen richness (PCA first axis: *P* value < 0.01, estimate = 0.20; research effort: *P* value < 0.01, estimate = 13.85; species range area: *P* value < 0.01, estimate = 0.25). The PCA second axis and number of species with overlapping ranges were significantly negatively correlated with pathogen richness (PCA second axis: *P* value < 0.01, estimate = − 0.26; overlapping ranges: *P* value < 0.01, estimate = − 0.23) (Fig. 5 and Supplementary Table S10). Research effort had the largest effect size.

### Model cross-validation

Machine learning models fitted with the original life history predictors had relatively similar RMSE scores with the models fitted with the PCA axes. The zero-inflated model fitted with the original life history predictors performed better when predicting new data (Supplementary Table S2). The machine learning models performed quite consistently regardless of the random split of the dataset. The zero-inflated models, on the other hand, had very high RMSE and performed inconsistently (Supplementary Table S2). Hence, we decided not to include the zero-inflated models in the ensemble model.

### Ensemble model predictions

**Figure 6:**
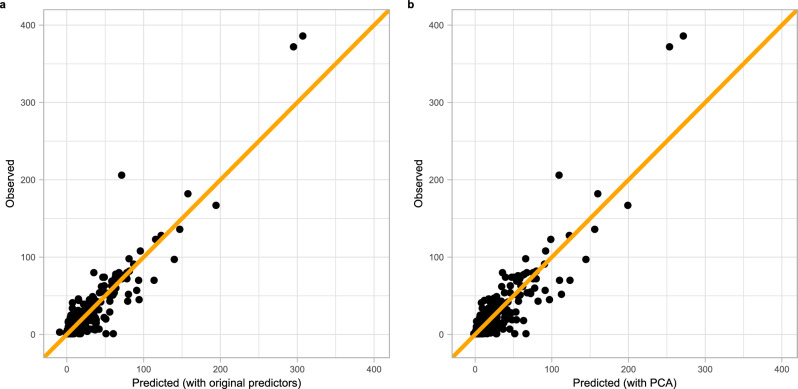
The predicted versus observed values of both ensemble models. (**a**) fitted with the original life history predictors; (**b**) fitted with the PCA axes. The line has an intercept of zero and slope of 1.

The predicted values from both ensemble models (fitted with the original life history predictors and with the PCA axes) tend to follow the observed data well, barring a few exceptions (Fig. 6). These exceptions were mainly from orders Carnivora and Artiodactyla (Supplementary Figs. S2 and S3). The ensemble model fitted with the original life history traits had a slightly better prediction accuracy (slope = 1.03, adjusted R^2^ = 0.89) than the ensemble model fitted with the PCA axes (slope = 1.03, adjusted R^2^ = 0.85).

Taxonomic orders Carnivora, Proboscidea, Artiodactyla, and Perissodactyla were predicted to host high pathogen richness (Supplementary Table S11). The top three species with the highest pathogen richness as predicted by the ensemble model fitted with the original life history predictors were the grey wolf (*Canis lupus*), wild boar (*Sus scrofa*), and moose (*Alces alces*). Similar results were found when the ensemble model was fitted with PCA axes representing the fast–slow continuum of life history traits (see “model_predictions.csv” in https://figshare.com/s/4acd0abbe58c7f1276a1 for all 5443 species predictions). However, when order Proboscidea was excluded, the red fox (*Vulpes vulpes*) was within the top three species instead of the moose (see “model_predictions_noele.csv” for the predictions without Proboscidea).

## Discussion

We found that pathogen richness in mammals was associated with research effort, order, species range area, and the fast–slow continuum of life history traits (neonate mass, weaning age, number of litters per year, and female maturity). Both extrinsic and intrinsic predictors played important roles, specifically species geographic distribution and species life history traits. Our results did not support our initial hypothesis; instead, they revealed that species with either low or high reproductive rates had higher pathogen richness. Furthermore, anthropogenic land-cover change did not show a clear relationship with pathogen richness. While urban land-cover change was positively related to pathogen richness, agricultural land-cover change yielded a negative relationship.

All models indicated a positive correlation between species range area and pathogen richness. Our findings are in line with previous results^11,19,38^, showing that geographic range area is a strong predictor for pathogen richness across different mammalian orders. Species with larger range area are exposed to more pathogens as they could potentially encounter more host species and different habitats^11^. An alternative explanation is that wide-ranging species are more likely to have higher pathogen richness because of geographic sampling bias^47^. Species with larger geographic ranges tend to be located at higher latitudes (i.e. Rapoport’s rule, but see^48^), where research effort is higher, probably due to greater resource allocation^47^. Furthermore, larger range areas are more likely to have overlaps with roads and urban areas, increasing the accessibility to these species. However, we argue that, if this is the case, our research effort variable should reflect a greater knowledge of species with greater geographic extents. In any case, future research on geographic sampling bias and known pathogen richness of a species is crucial to untangle the relationship between species range area and pathogen richness.

The fast–slow continuum of life history traits seems to have two pathways by which they accumulate pathogen richness. Our results showed that there is a duality to the relationship of fast–slow lived species and pathogen richness, where pathogen richness increases near the extremities (when the species has a very fast or very slow pace of life). The random forest model fitted with the original life history predictors showed that at very short weaning age, female maturity age, and higher number of litters per year (fast-lived), there was high pathogen richness. As female maturity age and weaning age increases and the number of litters per year decreases (slow-lived), pathogen richness decreases. This could be because of the reproduction and immunity trade-offs, where slow-lived species have greater energy investment in immunity^24^ resulting in less pathogen richness. After the point of inflection, the increase in pathogen exposure due to increased survival and life span increases pathogen richness^13,25^, as slow-lived species are more likely to being infected by a wide diversity of pathogens multiple times. This results in a U-shaped like relationship between the fast–slow continuum of life history traits and pathogen richness. This relationship was still observed after summarizing the fast–slow continuum of life history traits with PCA.

Land-cover change (agricultural land-cover change and urban land-cover change) had no clear trend with pathogen richness. Agricultural land-cover change had a negative correlation with pathogen richness while urban land-cover change had a positive correlation. Anthropogenic land-cover change has a complex and variable effect on host species communities and disease transmission^49,50^. The directional effect (whether positive or negative) of anthropogenic land-cover change on pathogen richness are compounded by various other factors such as the level of disturbance, temporal change, and number of host species present^49^. Hence, understanding the relationship between anthropogenic land-cover change and pathogen richness may require local-based predictors.

The taxonomic orders predicted to host high average pathogen richness by our ensemble models were Carnivora, Proboscidea, Artiodactyla, and Perissodactyla while previous studies based on current empirical pathogen data identified orders Rodentia, Chiroptera, Primata, Artiodactyla, Perissodactyla, and Carnivora to be major zoonotic disease reservoirs^17,51^. Although there are some overlaps, Rodentia and Chiroptera were not the top few orders with high predicted pathogen richness while Proboscidea was predicted to have high pathogen richness. Rodentia and Chiroptera are large mammalian orders that are widely distributed, with high species richness^17^, and commonly found in urban environments^31^. Hence, even though they host a lower pathogen richness on average, they are still important reservoirs. It is also important to note that some orders comprise of only a few extant species. This might lead to poor estimation of the effect of the order. Relatedly, estimation at the extreme end of the slow-lived continuum corresponds to a few species of the order Proboscidea which may lead to unreliable estimates at this end of the fast–slow continuum. For instance, when Proboscidea was removed from the analysis, pathogen richness for very slow-lived species decreased (averaged pathogen richness for *Alces alces* decreased from 206 to 103). Removing Proboscidea, although affects estimates for extreme species, did not change the results where the U-shaped relationship between pathogen richness and fast–slow continuum of life history traits remained.

Surveillance of pathogen richness in wildlife is essential to prevent zoonotic disease spillover and identify possible areas of zoonotic emerging infectious diseases. However, such monitoring requires a large amount of time and resources, making it challenging to employ throughout understudied areas such as the tropics^52^. Our ensemble model can predict pathogen richness for species with missing pathogen data. This allows identification of species that probably harbor high pathogen richness but are currently understudied. This knowledge combined with other factors of zoonotic disease spillover risk can help to identify key areas and species for surveillance to prevent emergence of future zoonotic diseases. For instance, wildlife markets increase the risk of zoonotic disease spillover due to the close human-wildlife interactions^53^. A local wildlife market in Northeast India was estimated to trade more than 3000 mammals annually and the wild meat were supplied by local indigenous hunters^54^. Some of these traded species, such as the orange-bellied Himalayan squirrel (*Dremomys lokriah*) and spotted linsang (*Prionodon pardicolor*), have low research effort and no pathogen richness data. Our ensemble models predicted the orange-bellied Himalayan squirrel and the spotted linsang to have an average pathogen richness of 42 and 95, respectively. These predictions can allow better understanding of the zoonotic disease risk of these understudied species.

Our results focused on the trade-offs between reproduction and immunity when considering the fast–slow continuum of life history traits, however, other non-reproductive life history traits may affect immunity^55,56^. For instance, bats have higher metabolic capacity which they have evolved for flight^55^. Their high metabolic rates may have allowed daily activation of the immune system, thus reducing the virulence of the virus and allowing them to be more tolerant of viral infections^56^. This allows bats to be asymptomatic carriers of a diversity of viruses^56,57^.

Our analysis was at the species level and thus, some of our predictors were at a macro scale with low spatial and temporal resolution. This results in limitations to our study as the models do not account for different populations hosting varying pathogen richness, host and vector species community interactions, and temporal variations in predictors and pathogen richness. Some possible predictors of pathogen richness (i.e., species metabolic rate and hunting pressure) were omitted from our analysis due to data limitations. Furthermore, as the models made predictions for species without pathogen richness data, these predictions cannot be validated and should be treated with caution. Thus, we call for further research toward neglected species to monitor potential disease reservoir and prevent the emergence of future zoonotic diseases.

## Conclusion

All models concur that species geographic range area is an important predictor and is positively correlated with pathogen richness. Moreover, our results show that there is no simple linear relationship between fast–slow continuum of life history traits and pathogen richness. Instead, there is a duality in the relationship where pathogen richness increases for very fast-lived and very slow-lived species. Our models can be used to guide searches for pathogen diversity in understudied systems which have the potential for zoonotic disease spillover. This aids in identifying mammalian species that should be prioritized for disease surveillance. Specifically, taxonomic orders Carnivora, Proboscidea, Artiodactyla, and Perissodactyla were predicted to host high pathogen richness.

### Supplementary Information

Supplementary Information.

## Data Availability

The data and codes used in this study are available at https://figshare.com/s/4acd0abbe58c7f1276a1.
