# Supplementary material for: Range area and the fast–slow continuum of life history traits predict pathogen richness in wild mammals
Source: Sci Rep. 2023 Nov 18;13:20191. doi: 10.1038/s41598-023-47448-3 (PMC10657380; doi:10.1038/s41598-023-47448-3)
Supplement: Supplementary file 1 — Supplementary Information. [file 41598_2023_47448_MOESM1_ESM.docx]

**Supplementary information**

Supplementary information for

Range area and the fast-slow continuum of life history traits predict pathogen richness in wild mammals

Choo et al.

Table S1. Predictors used in our models with their expected relationship with pathogen richness, data source, and percentage of imputation.

| Predictors | Hypothesis | Data source | Imputation percentage (%) |
| --- | --- | --- | --- |
| Species geographic distribution | | | |
| Geographic range area (km^2^) | Species with larger range size/altitude breadth may occupy more different habitats and have higher pathogen exposure, which can lead to higher pathogen richness^[1]^.  Species with larger range area and altitude breadth have higher pathogen richness. | IUCN extant range^[2]^ | 0 |
| Altitude breadth (m) |  | COMBINE database^[3]^ | 53.5 |
| No. of species with overlapping ranges | Species with a higher number of species whose ranges overlap may come into contact with other species more frequently. This increases the species exposure to pathogens and other host species, leading to higher pathogen richness^[1,4]^.  The greater number of species whose ranges overlap with the target species, the higher pathogen richness of the target species. | IUCN extant range^[2]^ | 0 |
| Anthropogenic factors | | | |
| Agricultural land-cover change | Species with greater anthropogenic land-cover change across its range have increase human-wildlife and domesticated animals-wildlife contact, increasing the risk of pathogen transmission. Higher anthropogenic land-cover change also results in increase environmental contaminants and the loss of habitats^[5,6]^, resulting in weaken immunity and higher pathogen richness.  Species with greater anthropogenic land-cover change across its range have higher pathogen richness. | ESA CCI Land Cover and the EC C3S Land cover project^[7]^ | 0 |
| Urban land-cover change |  |  |  |
| Livestock density (n/km^2^) | Greater exposure to livestock can lead to pathogen transmission/spillback from domesticated animals to wildlife^[8,9]^.  Species with greater livestock density across its range have higher pathogen richness. | FAO (2015)^[10]^ | 0 |
| Species traits | | | |
| Maximum longevity (days) | Due to the trade-offs between reproduction and immunity, fast-lived species have lower investment in immunity. This can lead to higher pathogen richness found in fast-lived species^[11,12]^.  Fast-lived species (shorter longevity, gestation length, female maturity, age at first reproduction, lower neonate mass, and weaning age) have higher pathogen richness. | COMBINE database^[3]^ | 54.5 |
| Gestation length (days) |  |  | 61.0 |
| Female maturity (days) |  |  | 65.0 |
| Age at first reproduction (days) |  |  | 65.3 |
| Neonate mass (g) |  |  | 66.4 |
| Weaning age (days) |  |  | 63.9 |
| Litter size | Fast-lived species (bigger litter size and higher number of litters per year) have higher pathogen richness. |  | 37.5 |
| No. of litters per year |  |  | 61.9 |

Table S2. The root-mean-squared error (RMSE) of all models.

| Models | RMSE of training data | RMSE of testing data | 10-fold cross validation |
| --- | --- | --- | --- |
| Range of observed pathogen richness: 1 to 372 | | | |
| Random forest with original predictors | 8.10 | 14.54 | 7.67 |
| Random forest with PCA | 8.46 | 15.29 | 7.79 |
| Boosted regression tree with original predictors | 4.62 | 13.98 | 10.61 |
| Boosted regression tree with PCA | 9.97 | 12.27 | 10.79 |
| Zero-inflated with original predictors | 3244.82 | 24.07 | 4.01·10^23^ |
| Zero-inflated with PCA | 2.32·10^15^ | 2.69·10^70^ | 9.73·10^40^ |

# **Random forest**

Table S3. Hyperparameter tuning results for bagged random forest. RMSE: root-mean-square error.

| Bagged random forest without PCA | | | Bagged random forest with PCA | | |
| --- | --- | --- | --- | --- | --- |
| mtry | RMSE | Rsquared | mtry | RMSE | Rsquared |
| 1 | 21.58 | 0.42 | 1 | 21.74 | 0.42 |
| 2 | 19.30 | 0.49 | 2 | 19.22 | 0.49 |
| 3 | 18.26 | 0.53 | 3 | 17.94 | 0.54 |
| 4 | 17.55 | 0.55 | 4 | 17.32 | 0.55 |
| 5 | 17.18 | 0.56 | 5 | 17.02 | 0.56 |
| 6 | 16.92 | 0.57 | 6 | 16.85 | 0.56 |
| 7 | 16.71 | 0.57 | 7 | 16.80 | 0.56 |
| 8 | 16.46 | 0.58 | 8 | 16.66 | 0.56 |
| 9 | 16.45 | 0.57 | 9 | 16.63 | 0.56 |
| 10 | 16.35 | 0.57 | 10 | 16.75 | 0.55 |
| 11 | 16.25 | 0.58 |  |  |  |
| 12 | 16.25 | 0.58 |  |  |  |
| 13 | 16.19 | 0.58 |  |  |  |
| 14 | 16.24 | 0.57 |  |  |  |
| 15 | 16.23 | 0.58 |  |  |  |
| 16 | 16.24 | 0.57 |  |  |  |

Table S4. The predictors importance in the random forest model with the original life history predictors (mtry = 13). The percentage increase in mean squared error (%IncMSE) shows the increase in the model error (decrease in the model accuracy) if the variable is excluded.

| Predictors | %IncMSE |
| --- | --- |
| Research effort | 33.32 |
| Order | 19.75 |
| Neonate mass (g) | 10.77 |
| Species range area (km^2^) | 9.82 |
| Weaning age (days) | 6.26 |
| Altitude breadth (m) | 5.89 |
| No. of litters per year | 5.04 |
| % of urban land-cover change (1992 – 2020) | 4.07 |
| Female maturity (days) | 3.95 |
| Gestation length (days) | 3.86 |
| % of agricultural land-cover change (1992 – 2020) | 2.14 |
| Max longevity (days) | 1.86 |
| No. of species with overlapping ranges | 1.70 |
| Age at first reproduction (days) | 0.84 |
| Litter size | 0.83 |
| Livestock density (n/km^2^) | 0.00 |

Table S5. The predictors importance in the random forest model fitted with PCA axes (mtry = 9). The percentage increase in mean squared error (%IncMSE) shows the increase in the model error (decrease in the model accuracy) if the variable is excluded.

| Predictors | %IncMSE |
| --- | --- |
| Research effort | 33.17 |
| Order | 23.38 |
| Species range area (km^2^) | 10.62 |
| PCA axis 1 | 5.96 |
| Altitude breadth (m) | 5.81 |
| % of agricultural land-cover change (1992 – 2020) | 3.86 |
| PCA axis 2 | 3.49 |
| No. of species with overlapping ranges | 3.33 |
| % of urban land-cover change (1992 – 2020) | 2.46 |
| Livestock density (n/km2) | -0.95 |

# **Boosted regression tree**

Table S6. Hyperparameter tuning results for the boosted regression tree. RMSE: root-mean-square error.

| shrinkage | Interaction depth | Bag fraction | Optimal trees | Minimum RSME |
| --- | --- | --- | --- | --- |
| Boosted regression tree without PCA | | | | |
| 0.1 | 5 | 1 | 290 | 20.15 |
| 0.01 | 5 | 1 | 3253 | 20.33 |
| 0.1 | 3 | 1 | 124 | 20.59 |
| 0.01 | 3 | 1 | 765 | 20.79 |
| 0.001 | 3 | 1 | 4199 | 21.35 |
| 0.01 | 3 | 0.8 | 2080 | 21.43 |
| 0.01 | 3 | 0.65 | 4982 | 21.68 |
| 0.01 | 5 | 0.65 | 4984 | 21.72 |
| 0.1 | 3 | 0.8 | 507 | 21.94 |
| 0.1 | 5 | 0.65 | 638 | 22.03 |
| Boosted regression tree with PCA | | | | |
| 0.1 | 3 | 0.8 | 214 | 21.22 |
| 0.01 | 3 | 0.8 | 2057 | 21.35 |
| 0.1 | 3 | 1 | 37 | 21.58 |
| 0.001 | 3 | 1 | 3682 | 21.72 |
| 0.01 | 3 | 1 | 368 | 21.73 |
| 0.1 | 3 | 0.65 | 183 | 21.78 |
| 0.01 | 3 | 0.65 | 2580 | 21.79 |
| 0.1 | 5 | 1 | 58 | 21.79 |
| 0.1 | 5 | 0.65 | 234 | 21.95 |
| 0.1 | 5 | 0.8 | 253 | 22.09 |

Table S7. The predictors importance in the boosted regression tree model without PCA. The relative influence shows the relative contribution of the explanatory variable to predicting mammalian pathogen richness.

| Predictors | Relative influence (%) |
| --- | --- |
| Research effort | 54.02 |
| Species range area (km^2^) | 19.74 |
| Neonate mass (g) | 7.57 |
| Order | 5.75 |
| Altitude breadth (m) | 3.40 |
| Litter size | 2.12 |
| No. of species with overlapping ranges | 1.26 |
| % of agricultural land-cover change (1992 – 2020) | 1.05 |
| % of urban land-cover change (1992 – 2020) | 1.04 |
| Gestation length (days) | 0.97 |
| Female maturity (days) | 0.97 |
| Max longevity (days) | 0.70 |
| Livestock density (n/km^2^) | 0.52 |
| No. of litters per year | 0.31 |
| Age at first reproduction (days) | 0.31 |
| Weaning age (days) | 0.26 |

Table S8. The predictors importance in the boosted regression tree model fitted with PCA axes. The relative influence shows the relative contribution of the explanatory variable to predicting mammalian pathogen richness.

| Predictors | Relative influence (%) |
| --- | --- |
| Research effort | 60.95 |
| Species range area (km^2^) | 19.07 |
| Order | 7.86 |
| Altitude breadth (m) | 4.40 |
| No. of species with overlapping ranges | 2.57 |
| PCA axis 2 | 2.01 |
| PCA axis 1 | 1.31 |
| Livestock density (n/km2) | 1.03 |
| % of agricultural land-cover change (1992 – 2020) | 0.49 |
| % of urban land-cover change (1992 – 2020) | 0.31 |

# **Zero-inflated negative binomial mixed-effects models**

Table S9. The zero-inflated negative binomial mixed-effects model with log-transformation. Selected predictors with right skewness were log-transformed and the rest of the predictors were standardized. All predictors were checked for multicollinearity (VIF < 5) and age at first reproduction was removed due to multicollinearity issue.

| Predictors | Estimate | Standard error | p-value |
| --- | --- | --- | --- |
| Intercept | -5.89 | 1.36 | < 0.01* |
| ln(Max longevity) | 0.40 | 0.10 | < 0.01* |
| ln(Gestation length) | -0.05 | 0.21 | 0.83 |
| ln(Female maturity) | -0.25 | 0.14 | 0.09 |
| ln(Weaning age) | 0.37 | 0.12 | < 0.01* |
| Altitude breadth | -0.07 | 0.06 | 0.20 |
| ln(Neonate mass) | 0.11 | 0.05 | 0.03* |
| ln(Litter size) | 0.43 | 0.17 | 0.01* |
| ln(No. of litters per year) | -0.26 | 0.17 | 0.14 |
| Research effort | 0.32 | 0.10 | < 0.01* |
| % of agricultural land-cover change (1992 – 2020) | -0.26 | 0.06 | < 0.01* |
| % of urban land-cover change (1992 – 2020) | 0.32 | 0.19 | 0.10 |
| ln(Species range area) | 0.23 | 0.03 | < 0.01* |
| Livestock density | -0.49 | 0.19 | 0.01* |
| No. of species with overlapping ranges | -0.21 | 0.07 | < 0.01* |

Table S10. The zero-inflated negative binomial mixed-effects model fitted with PCA axes using the all tropical species dataset. All predictors, with the exception of the PCA axes, were standardized. All predictors were also checked for multicollinearity (VIF < 5).

| Predictors | Estimate | Standard error | p-value |
| --- | --- | --- | --- |
| Intercept | -2.47 | 0.39 | < 0.01* |
| PCA axis 1 | 0.20 | 0.03 | < 0.01* |
| PCA axis 2 | -0.26 | 0.08 | < 0.01* |
| Altitude breadth | -0.10 | 0.06 | 0.10 |
| Research effort | 13.85 | 1.83 | < 0.01* |
| % of agricultural land-cover change (1992 – 2020) | -0.18 | 0.06 | < 0.01* |
| % of urban land-cover change (1992 – 2020) | -0.02 | 0.09 | 0.84 |
| ln(Species range area) | 0.25 | 0.03 | < 0.01* |
| Livestock density | -0.11 | 0.16 | 0.50 |
| No. of species with overlapping ranges | -0.23 | 0.07 | < 0.01* |

Table S11. The average pathogen richness for each taxonomic order. The + values are the standard error of mean.

| Orders | Number of species in observed data | Averaged observed pathogen richness | Number of species in predicted data | Averaged predicted pathogen richness | |
| --- | --- | --- | --- | --- | --- |
|  |  |  |  | Fitted with original life history predictors | Fitted with PCA axes |
| Artiodactyla | 123 | 22.6 + 3.9 | 231 | 116.7 ± 1.5 | 124.9 ± 1.5 |
| Carnivora | 117 | 22.9 + 4.2 | 253 | 87.2 ± 1.9 | 112.3 ± 1.9 |
| Chiroptera | 204 | 3.0 + 0.2 | 1240 | 34.1 ± 0.3 | 42.7 ± 0.3 |
| Cingulata | 5 | 2.6 + 1.4 | 20 | 47.4 ± 2.3 | 61.4 ± 2.3 |
| Dasyuromorphia | 4 | 1.3 + 0.3 | 71 | 38.1 ± 0.9 | 57.2 ± 0.9 |
| Didelphimorphia | 12 | 4.2 + 1.2 | 96 | 36.5 ± 0.8 | 55.0 ± 0.8 |
| Diprotodontia | 30 | 4.3 + 1.1 | 138 | 41.8 ± 0.7 | 61.2 ± 0.7 |
| Eulipotyphla | 14 | 3.4 + 1.2 | 469 | 37.2 ± 0.5 | 55.4 ± 0.5 |
| Hyracoidea | 1 | 2.0 | 5 | 70.9 ± 2.3 | 87.3 ± 2.3 |
| Lagomorpha | 17 | 7.4 + 4.6 | 93 | 55.0 ± 1.4 | 69.0 ± 1.4 |
| Monotremata | 2 | 1.5 + 0.5 | 5 | 77.4 ± 10.9 | 73.3 ± 10.9 |
| Peramelemorphia | 3 | 1.7 + 0.7 | 19 | 41.4 ± 1.3 | 60.6 ± 1.3 |
| Perissodactyla | 9 | 18.2 + 7.0 | 16 | 99.2 ± 2.2 | 105.5 ± 2.2 |
| Pilosa | 6 | 3.8 + 0.5 | 10 | 63.0 ± 5.9 | 62.5 ± 5.9 |
| Primates | 191 | 11.9 + 1.2 | 484 | 53.9 ± 0.6 | 62.3 ± 0.6 |
| Proboscidea | 2 | 8.0 + 1.0 | 2 | 95.8 ± 0.1 | 82.2 ± 0.1 |
| Rodentia | 298 | 4.4 + 0.6 | 2268 | 40.9 ± 0.2 | 52.5 ± 0.2 |
| Scandentia | 2 | 3.0 + 1.0 | 23 | 30.0 ± 1.6 | 46.3 ± 1.6 |

Table S12. The top 20 species prediction results of the uncertainty analysis with order Proboscidea removed. Full species list can be found in “model_predictions_noele.csv” in <https://figshare.com/s/4acd0abbe58c7f1276a1> for all 5441 species predictions).

| Ensemble model prediction results | | | Ensemble model prediction results with order Proboscidea removed | | |
| --- | --- | --- | --- | --- | --- |
| Species | Original life history predictors | PCA axes | Species | Original life history predictors | PCA axes |
| *Canis lupus* | 275.8 | 227.6 | *Canis lupus* | 173.8 | 166.3 |
| *Sus scrofa* | 269.2 | 240.7 | *Vulpes vulpes* | 161.6 | 157.2 |
| *Alces alces* | 208.8 | 203.3 | *Sus scrofa* | 152.6 | 138.0 |
| *Mellivora capensis* | 207.7 | 198.7 | *Mus musculus* | 137.3 | 130.4 |
| *Vulpes vulpes* | 194.8 | 200.3 | *Ursus arctos* | 130.6 | 119.4 |
| *Hyaena hyaena* | 192.0 | 179.3 | *Mustela erminea* | 127.7 | 120.8 |
| *Ursus arctos* | 189.9 | 178.6 | *Lutra lutra* | 125.9 | 118.8 |
| *Puma concolor* | 186.2 | 169.8 | *Mustela nivalis* | 125.6 | 122.2 |
| *Lutra lutra* | 175.0 | 178.8 | *Mellivora capensis* | 120.2 | 118.8 |
| *Ursus maritimus* | 169.7 | 170.9 | *Cervus elaphus* | 116.2 | 108.0 |
| *Rangifer tarandus* | 168.4 | 154.2 | *Gulo gulo* | 113.6 | 107.2 |
| *Lynx lynx* | 168.2 | 163.8 | *Hyaena hyaena* | 113.1 | 107.2 |
| *Mustela nivalis* | 167.8 | 186.8 | *Ursus maritimus* | 109.8 | 101.6 |
| *Mustela erminea* | 160.7 | 184.7 | *Lynx lynx* | 109.5 | 98.1 |
| *Gulo gulo* | 160.2 | 165.5 | *Lama guanicoe* | 109.2 | 102.2 |
| *Cervus elaphus* | 158.3 | 160.5 | *Rattus rattus* | 108.8 | 102.5 |
| *Sus celebensis* | 152.3 | 134.5 | *Alces alces* | 107.9 | 97.5 |
| *Sus philippensis* | 150.9 | 135.6 | *Cervus canadensis* | 106.7 | 97.4 |
| *Lontra canadensis* | 149.1 | 155.6 | *Cervus hanglu* | 106.4 | 97.6 |
| *Cervus hanglu* | 148.8 | 152.0 | *Puma concolor* | 106.3 | 95.9 |

# **Figures**


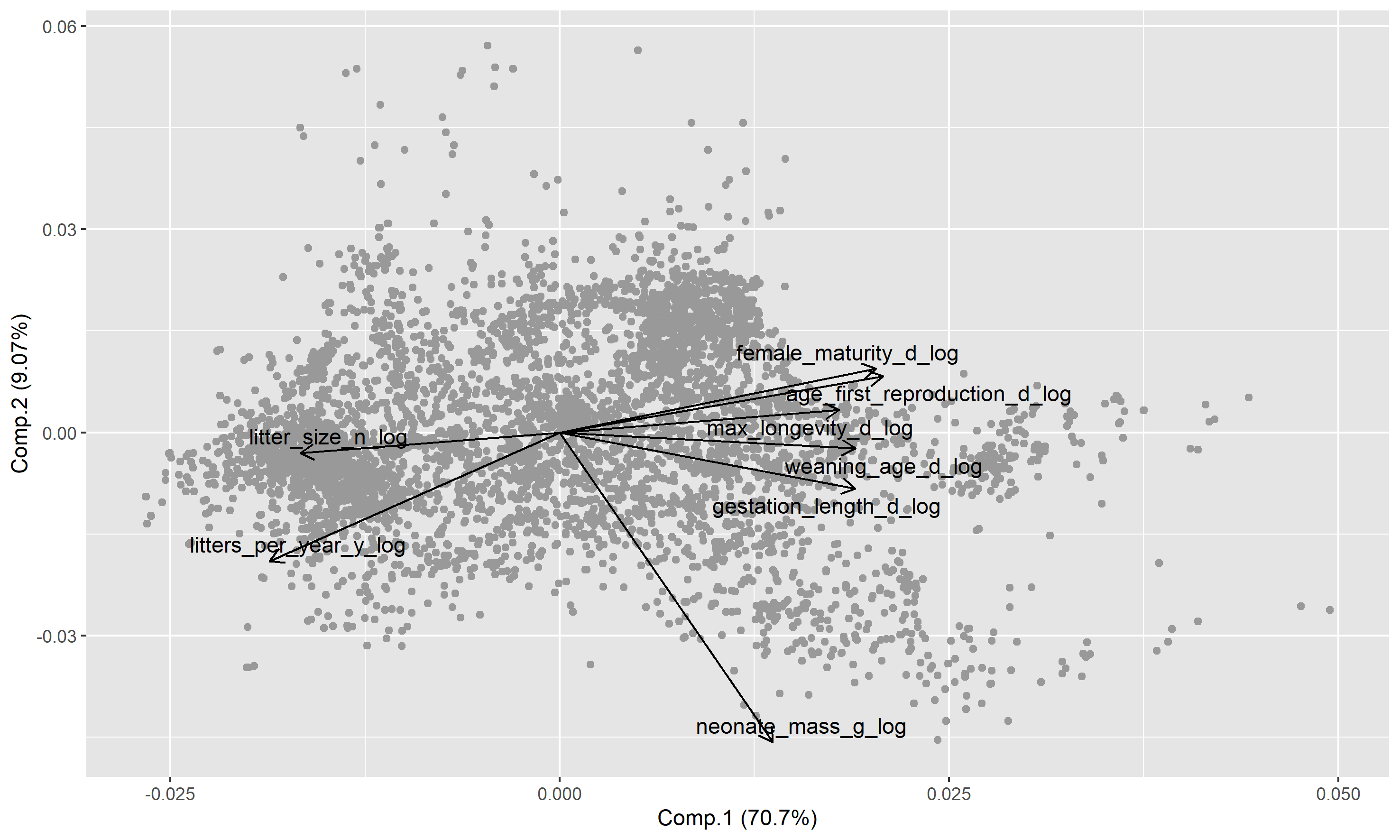


Figure S1. The principal component analysis (PCA) of the species fast-slow continuum of life history traits used in this study. The first axis (Comp.1) explained 70.7% of the variance while the second axis (Comp.2) explained 9.1%.


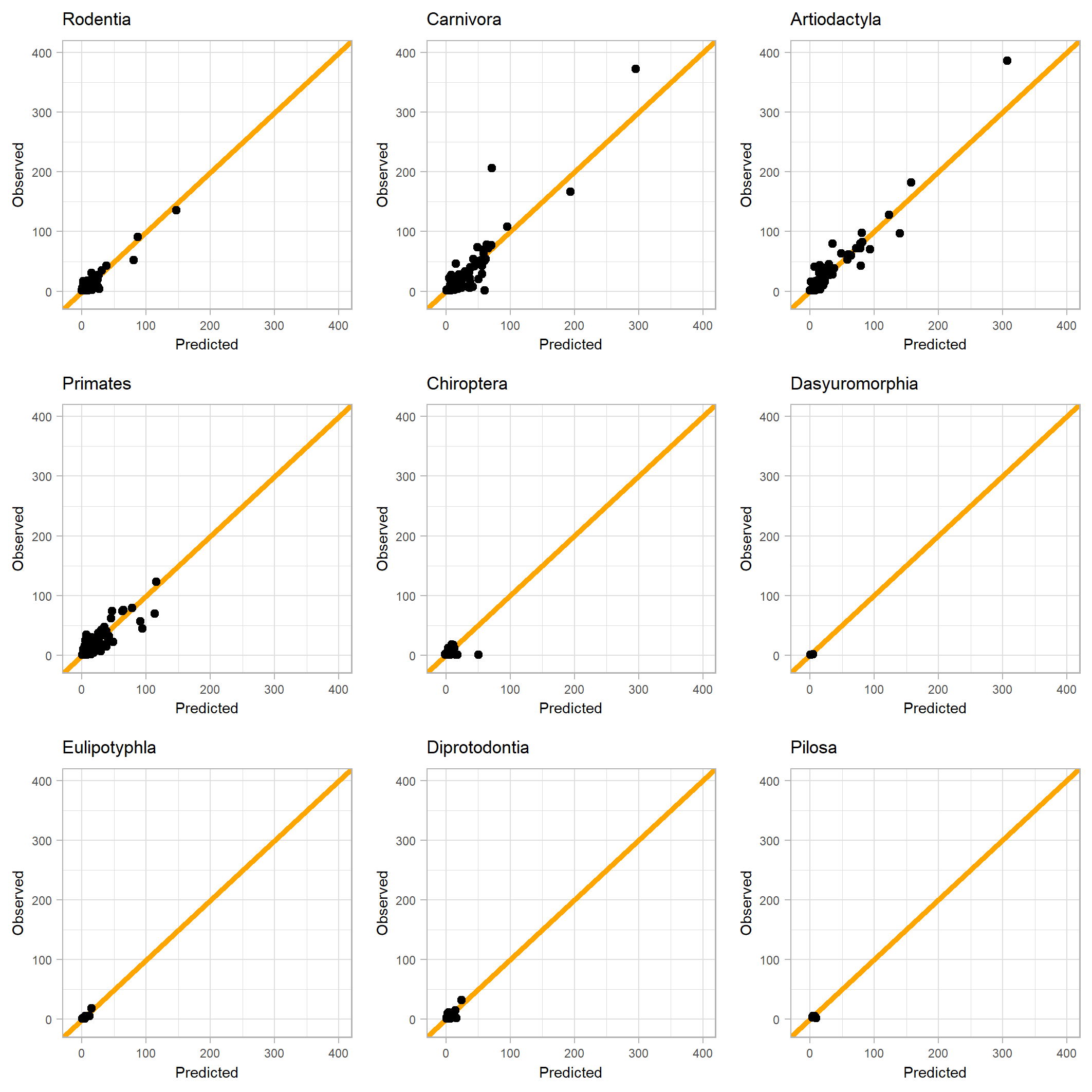


Figure S2. The predicted versus observed values of the ensemble model using the original predictors grouped by taxonomic orders.


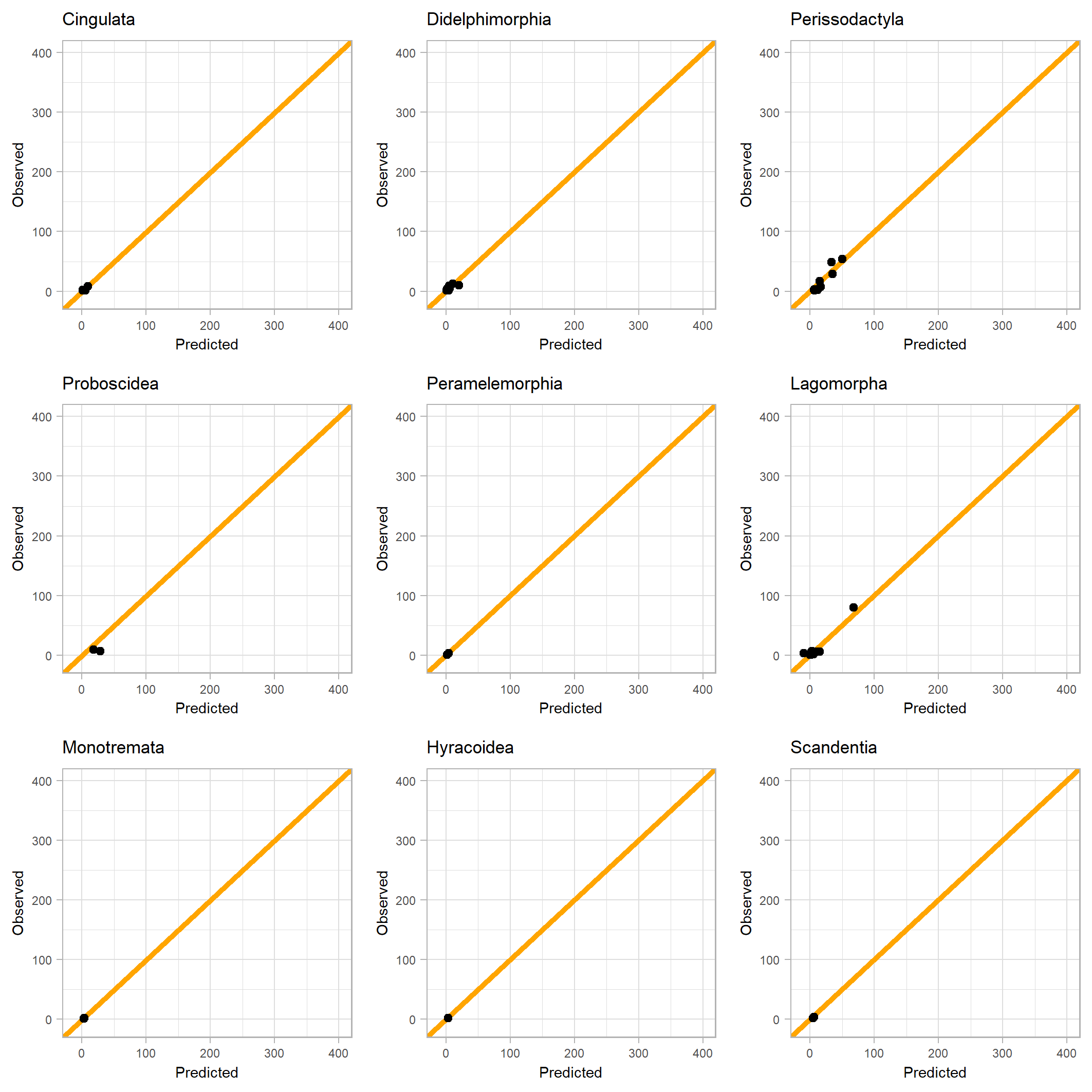


Figure S2 (continued). The predicted versus observed values of the ensemble model using the original predictors grouped by taxonomic orders.


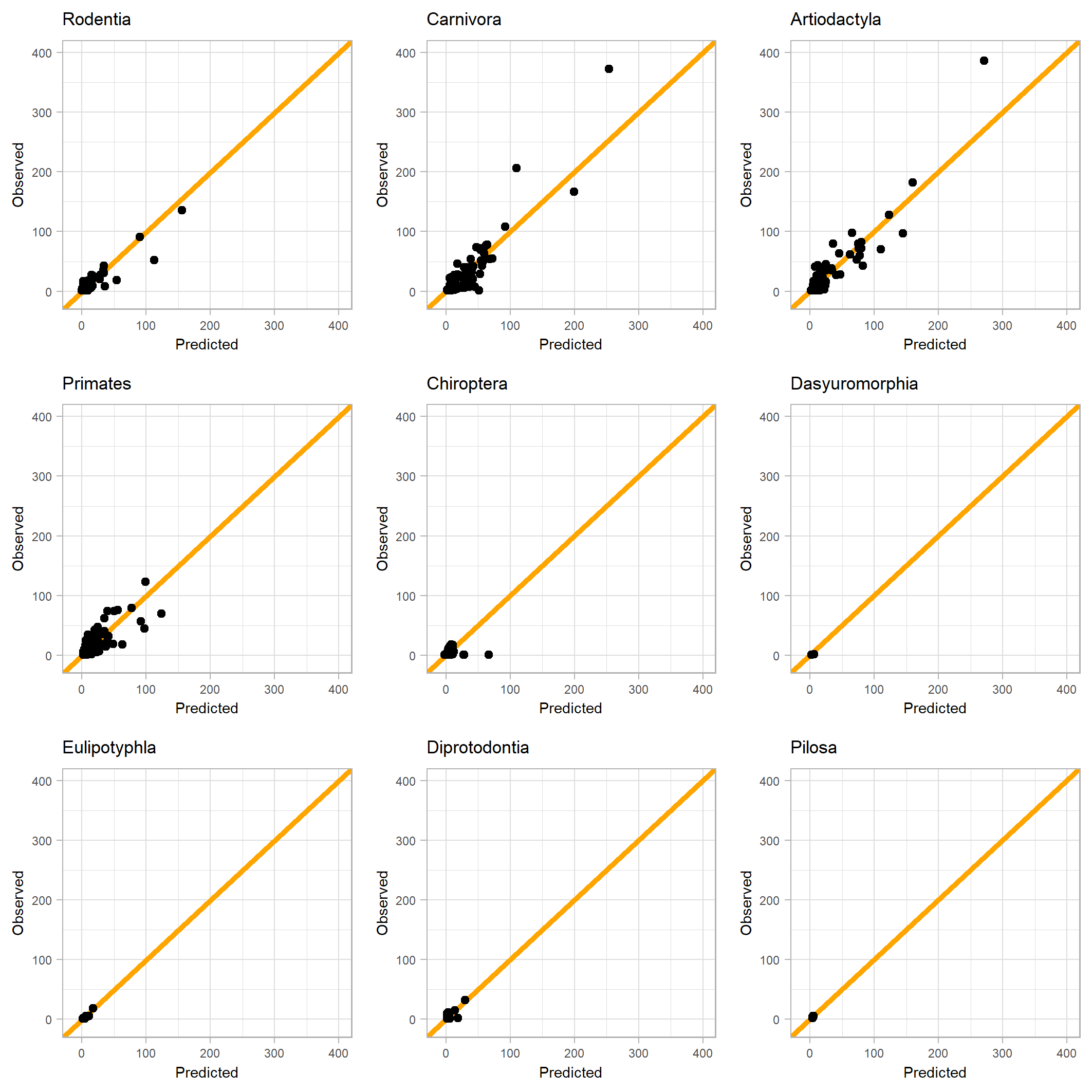


Figure S3. The predicted versus observed values of the ensemble model using PCA axes grouped by taxonomic orders.


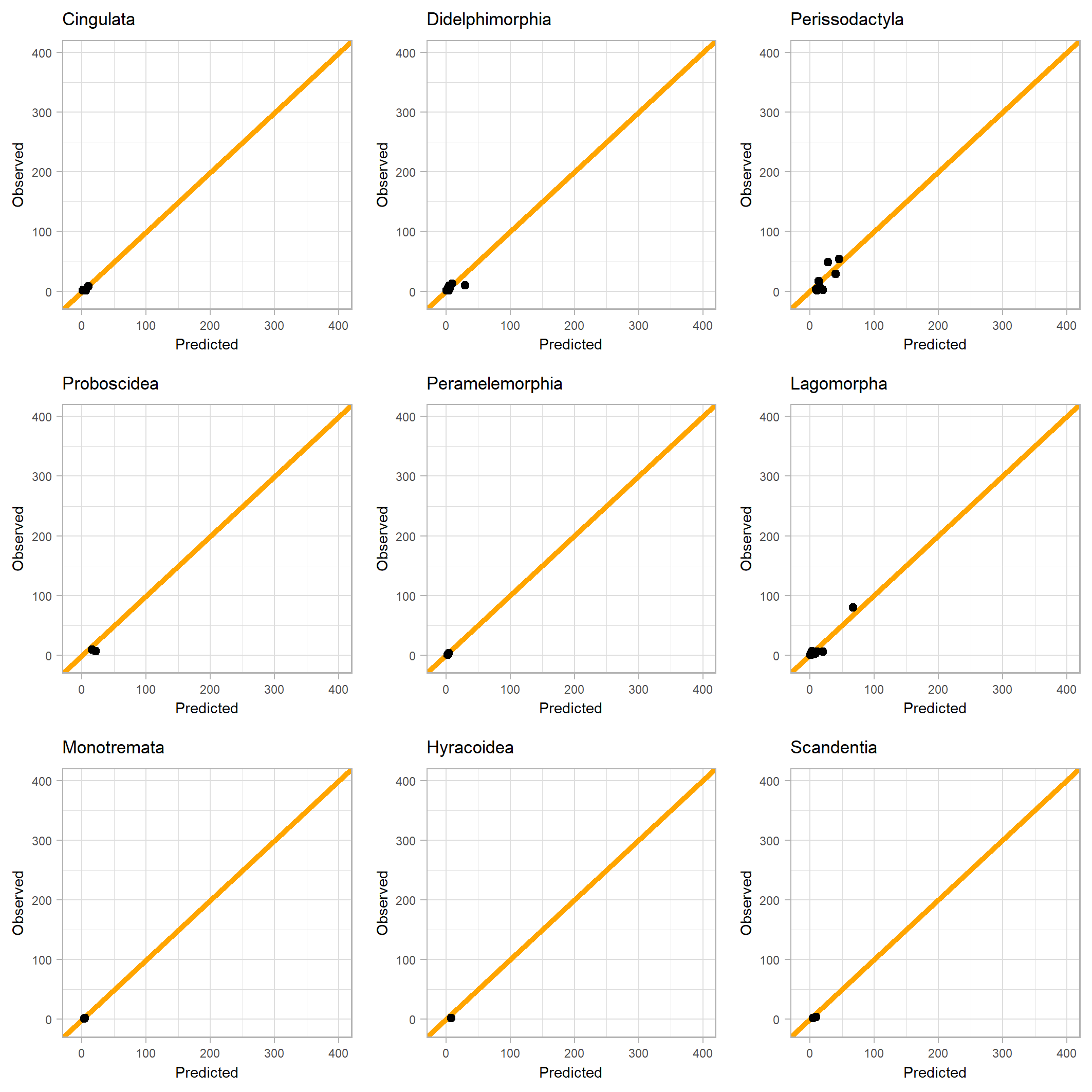


Figure S3 (continued). The predicted versus observed values of the ensemble model using PCA axes grouped by taxonomic orders.


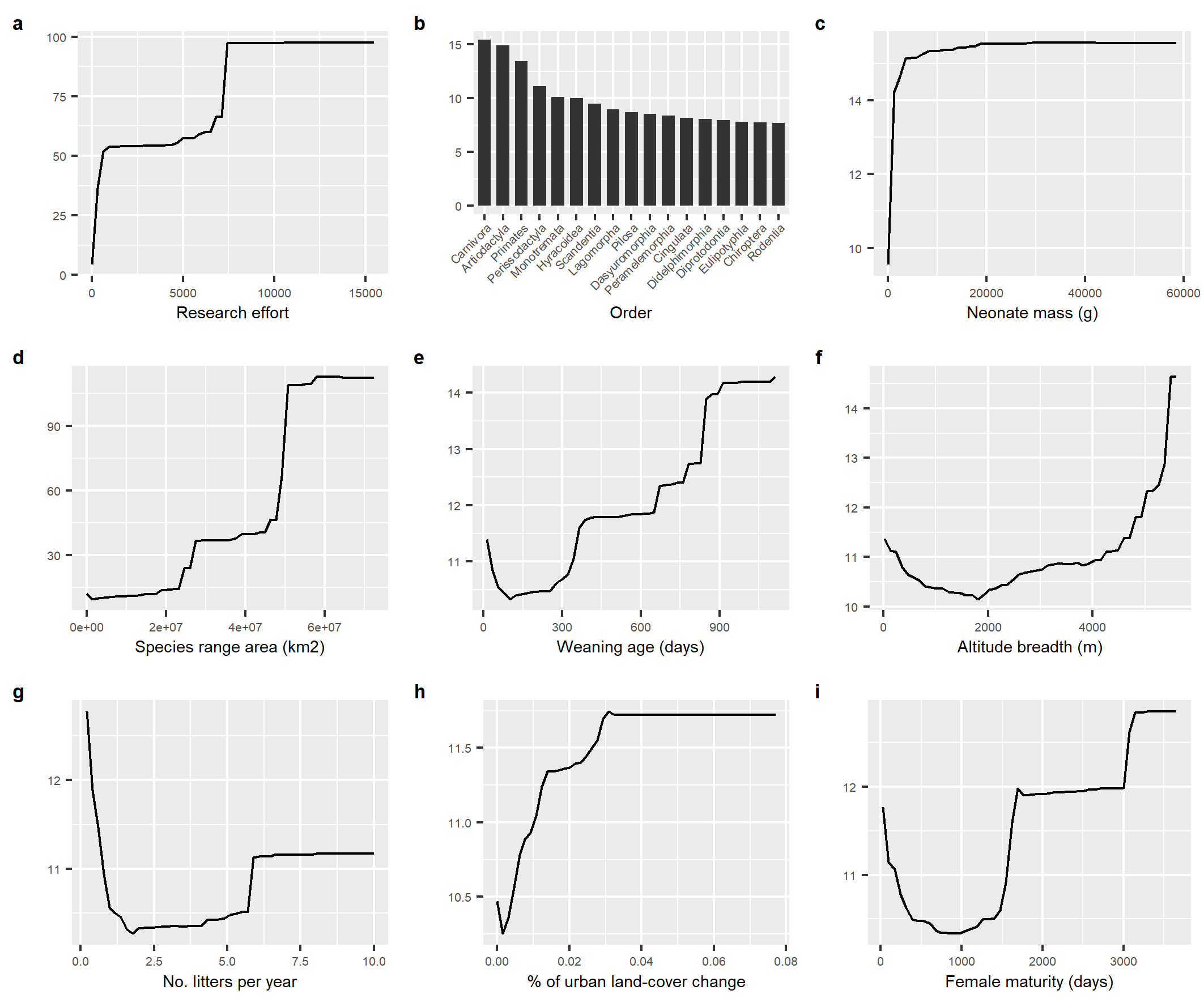


Figure S4. The partial dependence plots of the random forest model with order Proboscidea removed. The plots are the top nine predictors of the model fitted with the original life history predictors.


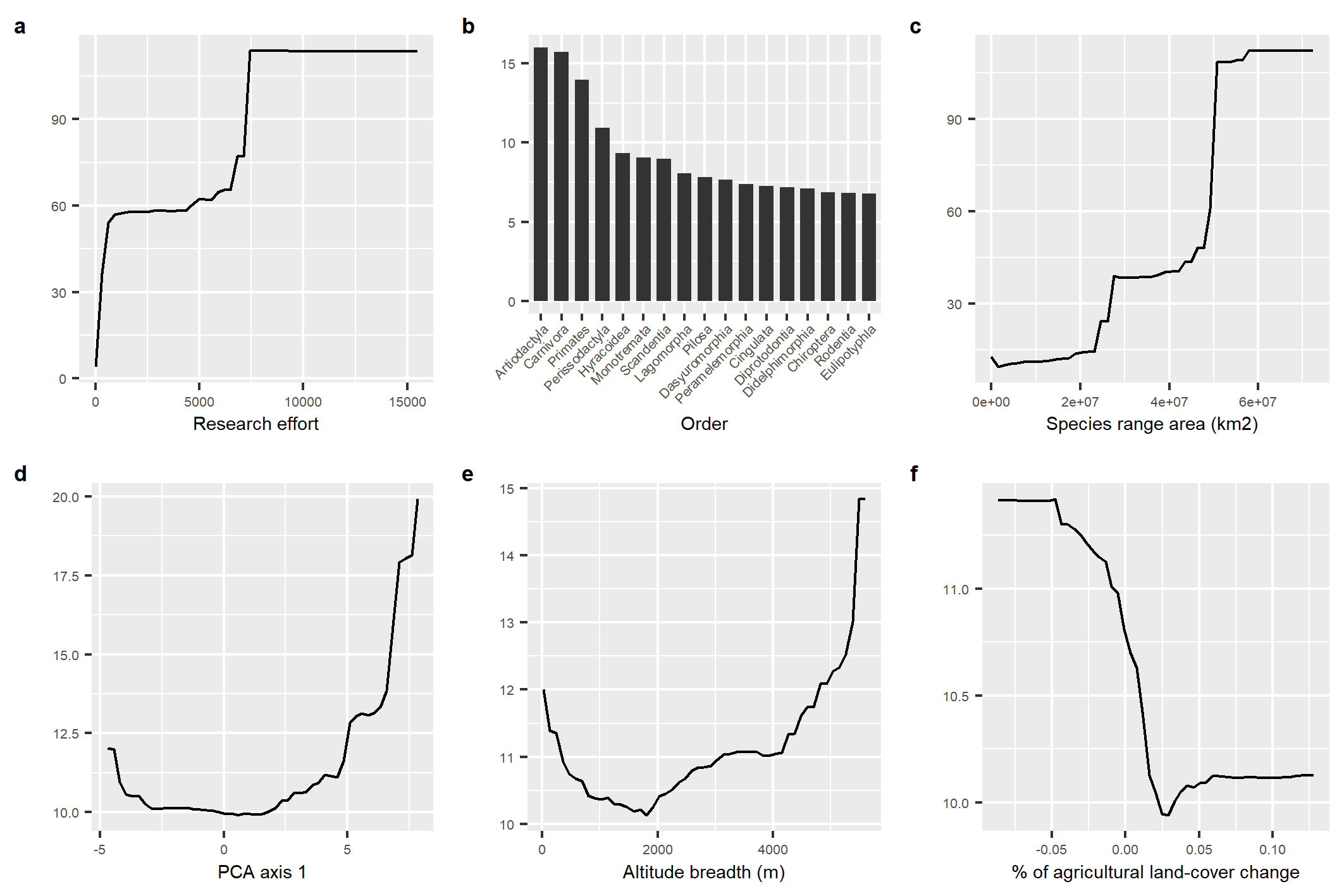


Figure S5. The partial dependence plots of the random forest model with order Proboscidea removed. The plots are the top six predictors of the model fitted with PCA axes. The negative values on PCA axis 1 are more fast-lived species and the positive values are more slow-lived species.

References:

1. Kamiya, T., O’Dwyer, K., Nakagawa, S. & Poulin, R. What determines species richness of parasitic organisms? A meta-analysis across animal, plant and fungal hosts: Determinants of parasite species richness. *Biol. Rev.* **89**, 123–134 (2014).

2. IUCN. Spatial Data Download. https://www.iucnredlist.org/resources/spatial-data-download.

3. Soria, C. D., Pacifici, M., Di Marco, M., Stephen, S. M. & Rondinini, C. COMBINE: a coalesced mammal database of intrinsic and extrinsic traits. *Ecology* **102**, (2021).

4. Olival, K. J. *et al.* Host and viral traits predict zoonotic spillover from mammals. *Nature* **546**, 646–650 (2017).

5. Becker, D. J. *et al.* Macroimmunology: The drivers and consequences of spatial patterns in wildlife immune defence. *J. Anim. Ecol.* **89**, 972–995 (2020).

6. Murray, M. H. *et al.* City sicker? A meta‐analysis of wildlife health and urbanization. *Front. Ecol. Environ.* **17**, 575–583 (2019).

7. Copernicus Climate Change Service. Land cover classification gridded maps from 1992 to present derived from satellite observations. (2019) doi:10.24381/CDS.006F2C9A.

8. VerCauteren, K. C., Lavelle, M. J. & Campa, H. Persistent Spillback of Bovine Tuberculosis From White-Tailed Deer to Cattle in Michigan, USA: Status, Strategies, and Needs. *Front. Vet. Sci.* **5**, 301 (2018).

9. Verheyden, H. *et al.* Relationship between the excretion of eggs of parasitic helminths in roe deer and local livestock density. *J. Helminthol.* **94**, e159 (2020).

10. FAO. Gridded Livestock of the World. https://www.fao.org/livestock-systems/global-distributions/en/.

11. Plourde, B. T. *et al.* Are disease reservoirs special? Taxonomic and life history characteristics. *PLOS ONE* **12**, e0180716 (2017).

12. Albery, G. F. & Becker, D. J. Fast-lived Hosts and Zoonotic Risk. *Trends Parasitol.* **37**, 117–129 (2021).
